# Evolution of Insect Pollination Before Angiosperms and Lessons for Modern Ecosystems

**DOI:** 10.3390/insects17010103

**Published:** 2026-01-16

**Authors:** Ilaria Negri, Mario E. Toledo

**Affiliations:** Department of Sustainable Crop Production (DI.PRO.VE.S.), Università Cattolica del Sacro Cuore, Via Emilia Parmense 84, 29122 Piacenza, Italy; ilaria.negri@unicatt.it

**Keywords:** pollination evolution, pre-angiosperm pollination, insect-gymnosperm pollination, insect-angiosperm mutualism, pollinator resilience, bee evolution, Anthropocene, plant–pollinator networks, pollinator health

## Abstract

Insect pollination, a critical ecological process, pre-dates the emergence of angiosperms by nearly 200 million years, with fossil evidence indicating pollination interactions between insects and non-angiosperm seed plants during the Late Paleozoic. This review examines the symbiotic relationships between insects and gymnosperms in pre-angiosperm ecosystems, highlighting the complexity of these interactions. Fossil records suggest that the mutualistic relationships between insects and gymnosperms, which facilitated plant reproduction, were as intricate and diverse as the modern interactions between angiosperms and their pollinators, particularly bees. These early pollination systems likely involved specialized behaviors and plant adaptations, reflecting a sophisticated evolutionary dynamic long before the advent of flowering plants. The Anthropocene presents a dichotomy: while climate change and anthropogenic pressures threaten insect biodiversity and risk disrupting angiosperm reproduction, such upheaval may simultaneously generate opportunities for novel plant–insect interactions as ecological niches are vacated. Understanding the deep evolutionary history of pollination offers critical insight into the mechanisms underlying the resilience and adaptability of these mutualisms. The evolutionary trajectory of bees—originating from predatory wasps, diversifying alongside angiosperms, and reorganizing after mass extinctions—exemplifies this dynamic, demonstrating how pollination networks persist and reorganize under environmental stress and underscoring the enduring health, resilience, and adaptability of these essential ecological systems.

## Simple Summary

Pollination by insects is one of the most important processes supporting life on Earth, allowing plants to reproduce and ecosystems to thrive. It is often thought that insect pollination began with flowering plants, but evidence from fossils shows that it started almost 300 million years earlier, when insects interacted with ancient seed plants long before flowers evolved. This review explores how those early relationships between plants and insects developed, changed, and survived major global crises such as mass extinctions and climate shifts. By looking at this long evolutionary history, we can better understand why pollination is such a resilient system and how it has adapted to past environmental challenges. These lessons from the deep past help us interpret what is happening today, as modern pollinators face threats from climate change, habitat loss, and human activities. Understanding how pollination networks have persisted for hundreds of millions of years can guide efforts to protect bees and other pollinators that are essential for both natural ecosystems and human food production.

## 1. Introduction

One of the main prejudices still rooted in the understanding of insect pollination is the belief that it began with the appearance and expansion of angiosperms in the late Lower Cretaceous. This process has long been attributed to a co-evolutionary relationship between flowering plants and pollinating insects, which was thought to have dramatically accelerated their biodiversity [1]. However, this view overlooks the extensive history of insect–plant mutual interactions that preceded angiosperms, with insect pollination predating the emergence of flowering plants (angiosperms) by nearly 200 million years.

Although the origin of insects is dated to the Early Ordovician [2], the first fossil evidence of insect taxa appears in the Early Devonian, somewhat later than the colonization of land by vascular plants. Even at this stage, clues suggest that spore consumption—i.e., feeding on the reproductive cells of early plants and fungi—was among the earliest feeding strategies of ancient hexapods [3]. Throughout their long evolutionary history, insects and plants have undergone multiple radiations, developing both mutualistic and antagonistic strategies. Interactions with plants increasingly involved reproductive organs, which insects exploited for valuable nutrients, inadvertently favoring plant outcrossing. Fossil evidence demonstrates such relationships as far back as the Upper Carboniferous, when early gymnosperms dominated terrestrial ecosystems alongside cryptogams [3,4,5,6].

These interactions further evolved during the Mesozoic era, often referred to as the “Age of Gymnosperms.” During this time, gymnosperms exhibited remarkable diversity—including shrubs, lianas, mangroves, succulents, fast-growing herbaceous plants, and palm- or dicot-like forms [7,8,9,10,11]. Insects and gymnosperms formed complex associations that were ecologically comparable to modern pollination systems, though involving now-extinct plant lineages and insect groups that today play little or no role as pollinators. These fossil interactions reveal a sophisticated evolutionary prelude to modern angiosperm pollination [9,12].

**Figure 1:**
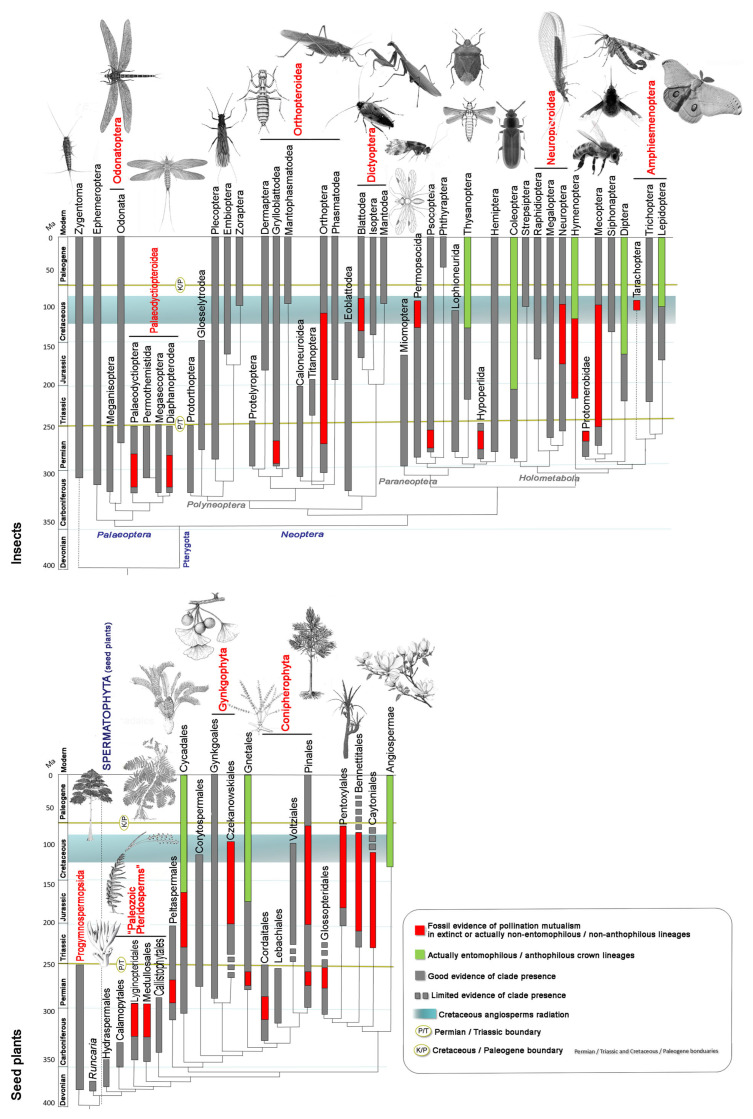
Schematic representation of the evolutionary history of insects (**top**) and seed plants (**bottom**). Highlighted (in red or green) are these lineages that include taxa known to have engaged in mutualistic pollination relationships, both in the fossil record and in modern ecosystems.

From the mid-Cretaceous onward (around 120 ma), gymnosperms faced increasing competition from emerging angiosperms, which progressively displaced them from many ecosystems [13]. In the modern era, gymnosperms persist as a “relictual” group, with several lineages occupying specialized ecological niches such as boreal forests (dominated by conifers) or tropical highlands (e.g., Cycadales). Figure 1 outlines the evolutionary history of plants and insects with reference to entomophily in plants and anthophily in insects, both of which appeared independently across multiple lineages over time.

The long-term history of insect pollination has been marked by major ecological disruptions and evolutionary turnovers. The Permian–Triassic and Cretaceous–Paleogene mass extinction events, rather than collapsing pollination systems, triggered extensive restructuring of insect–plant associations. These transitions included the replacement of declining Paleozoic paleopteran pollinators by neopterans—a process already underway before the Permian–Triassic crisis—and the reorganization of pollination networks during the Albian–Aptian angiosperm radiation. During this latter phase, specialized Mesozoic holometabolous pollinators declined in prominence as modern groups diversified, and some pollinators shifted from gymnosperms to angiosperms roughly 60 million years before the Cretaceous–Paleogene extinction [14,15].

Despite this evidence, the notion that complex mutualistic interactions between entomophilous plants and specialized pollinating insects predate the emergence of flowering plants remains underappreciated. This limited awareness reinforces the misconception that the ongoing ecological crisis of the Anthropocene will inevitably lead to the collapse of pollination—a mechanism that has sustained biodiversity for more than 300 million years and persisted through multiple episodes of profound environmental change.

Bees represent a paradigmatic example of such evolutionary turnover and adaptation. Originating from carnivorous, wasp-like ancestors in the Early Cretaceous, they underwent a profound ecological transition to pollen and nectar feeding. They subsequently diversified alongside flowering plants, reshaping pollination networks and establishing many of the interactions that underpin modern ecosystems [1,16,17]. Their trajectory highlights how pollinator lineages can rapidly radiate in response to plant innovations, reinforcing the long-term adaptability of plant–insect relationships [15,18].

Accordingly, this review provides a framework for understanding the evolutionary history of insect pollination, focusing on mutual relationships in pre-angiosperm ecosystems and the key steps leading to modern plant–pollinator systems. The angiosperm radiation marked a critical turning point, driving the diversification of pollinator lineages and promoting the coevolution of traits that sustain modern interactions—bees standing as the most prominent and emblematic example of this evolutionary partnership. Finally, we emphasize how this deep-time history demonstrates the inherent resilience of pollination systems, offering valuable insights into how they may respond to the unprecedented challenges of the Anthropocene.

## 2. Fossil Evidence of Host-Plant and Insect Interactions and the Evolutionary Phases of Insect Pollination

Past interactions between host plants and insects are well represented in the fossil record, as evidenced by various feeding modes on plant tissues [19]. Fossil evidence of entomophily reveals several factors comparable to those observed in modern pollination systems, such as the interplay between insect structures—especially mouthparts, but also other body parts like pollen baskets on the legs—adapted for collecting pollen and/or nectar, and the corresponding plant reproductive structures. Additional indicators include the presence of attractive floral features (e.g., nectar glands, showy organs), the morphology of pollen grains, and the occurrence of pollen associated with fossilized insect bodies or preserved within their coprolites [3,6,7,9,18,20,21,22,23,24,25,26,27]. The habit of a pollinator in a fossil insect could be also supposed if it belongs to a crown group of monophyletic lineages of extant taxa known as pollinators (e.g., Corbiculata bees) [28,29]. Finally, palaeoecological evidence of the environmental context of the possible plant-insect association is important for revealing feeding types and pollination modes that may be extinct or have survived to the present [6,20,30]. Actually, there is general acceptance that the application of these criteria in fossil records shows interactions between seed plants and insect pollinators, predating early angiosperm pollination by more than 200 million years, before the radiation of flowering plants, with associations ecologically similar in complexity to modern ones, especially during the Mesozoic [3,7,9,12,15,24,31]. An example of such associations can still be seen today in the obligate mutualistic relationship between Cycadales, the most ancient seed-plants living today, and their pollinating insects, which are certainly as complex as other species-specific relationships existing in various angiosperms [32,33].

**Figure 2:**
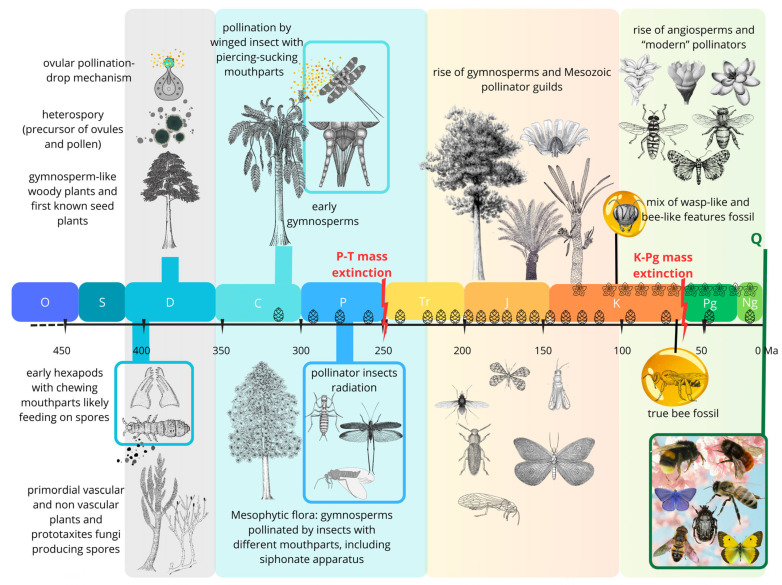
Schematic representation of the four major phases of plant and herbivorous insect expansion over the past 420 million years, from early arthropods associated with primitive vascular plants to the establishment of modern pollination networks in angiosperms. Each phase is distinguished by a different background color. The first two phases document the transition from spore-feeding and generalized herbivory to increasingly specialized insect interactions with seed plants, establishing the foundations of insect-mediated pollination prior to the origin of flowering plants. The third phase reflects the diversification of gymnosperms and the development of complex pollinator guilds involving multiple insect lineages exploiting reproductive structures. The fourth phase marks the radiation of angiosperms, the origin and diversification of bees, and the long-term resilience and reorganization of pollination networks to the present.

Fossils tell a 420-million-year-long story of vascular-plant hosts, their insect herbivores, and associated functional feeding groups, including palynivory and nectarivory, which were also involved in plant fecundation.This long story can be sorted spatiotemporally into four major herbivore expansions [19], discussed in detail in the subsequent paragraphs and summarized in Figure 2.

1. A Late Silurian to Late Devonian phase (about 60 million years) characterized by herbivorous arthropods, including apterygote hexapods (Entognathate and perhaps early Ectognathate), feeding on several clades of primitive vascular-plant hosts.
2. A Late Mississippian to end-Permian phase (85 million years) involving principally apterygotes, paleopterans, non-holometabolan and (later) basal holometabolan neopterans, feeding on pteridophytes, basal and more advanced gymnosperm plant hosts.
3. A Middle Triassic to Middle Cretaceous phase (ca. 130 million years) dominated by polyneopterans, paraneopterans and holometabolous, feeding mostly on gymnosperm plant hosts.
4. A mid-Early Cretaceous to Recent phase (115 million years) featuring modern hemimetabolous and holometabolous, feeding principally on angiosperm plant hosts. This phase also witnessed the emergence of bees, establishing one of the most important modern pollinator lineages.

While the underlying drivers of these four major associations are still debated, they likely reflect broad paleoclimatic and atmospheric dynamics, including greenhouse–icehouse cycles and fluctuations in O_2_ and CO_2_ concentrations. Notably, although the specific plant and arthropod taxa involved have changed through time, the fundamental feeding strategies have remained remarkably conserved across much of this evolutionary continuum.

### 2.1. Early Mandibles on Early Spores: Silurian-Devonian First Evidence of Palynivory

The first radiation of primordial vascular plants on land during the Silurian-Devonian period, alongside nonvascular plants and prototaxites fungi, led to early associations with ancient terrestrial arthropods, whose presence is documented in fossil deposits from that era [3,34,35,36]. Late Silurian and Early Devonian coprolites, i.e., fossilized faecal pellets, provide evidence of consumption by herbivorous arthropods of various organic materials, including spores [36,37]. Faecal pellets were likely produced by different groups of arthropods, potentially including early Entognathate hexapods such as the collembola species *Rhyniella praecursor* Hirst & Maulik, 1926, one of the oldest known hexapods dating back to the Early Devonian period, and possibly basal Ectognathate wingless insects [3,9]. According to Labandeira [3,5,7,19] and Labandeira et al. [9], these Late Silurian-Early Devonian interactions represent the earliest phase in the development of palinivory (i.e., the consumption of spores and pollen) and related feeding strategies, though at this stage, only spore consumption occurred. At that time, wind and water were the primary vectors for the dispersal of undifferentiated spores from these early tracheophytes, similar to the mechanisms used by extant ferns and horsetails, with no evidence of animal involvement in their reproduction or dispersal [13].

### 2.2. Seed Plants and the Second Phase of Plants/Insect Associations: Late Paleozoic Pollination

The Late Paleozoic marks the second major phase of insect exploitation of plant reproductive structures and likely the origin of plant–pollinator interactions, laying the groundwork for the complexity developed in the Mesozoic [7,9].

**Figure 3:**
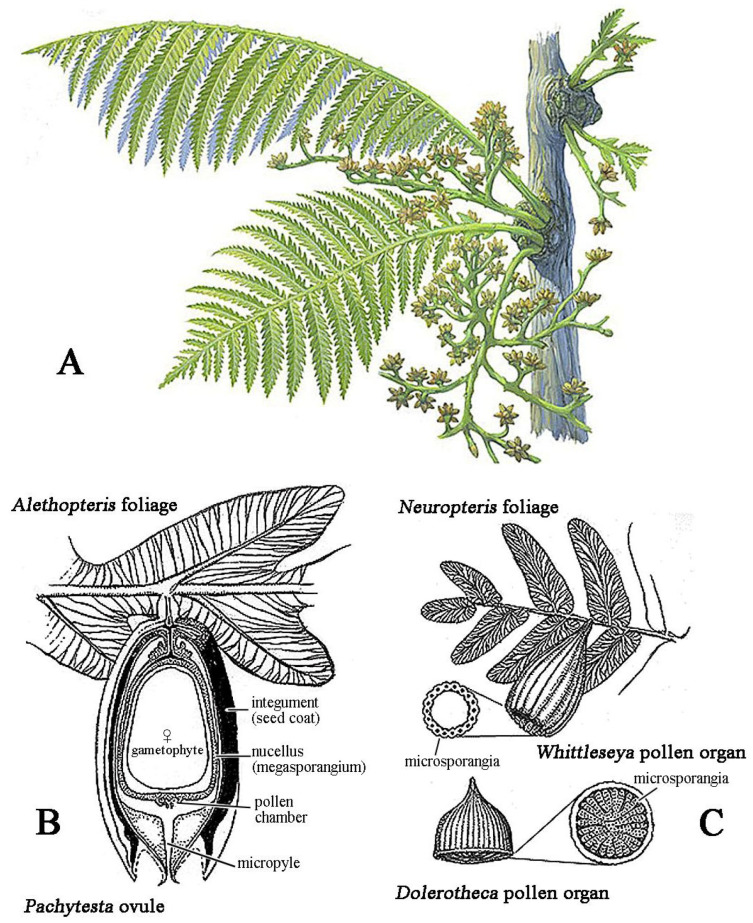
Paleozoic early seed plants. (**A**) Restoration of a Peltaspermales branch with foliage and male reproductive organs (modified from [29], CC BY 4.0; illustration by Michael Rothman; via Wikimedia Commons: https://commons.wikimedia.org/w/index.php?curid=138062109; accessed on 15 September 2025). (**B**,**C**) Medullosales reproductive organs: (**B**) Ovule of *Pachytesta* attached to *Alethopteris* foliage; (**C**) pollen organs (*Whittleseya*) attached to *Neuropteris* foliage and, below, the structure of *Dolerotheca* showing a complex system of pollen sacs (modified from: https://ucmp.berkeley.edu; accessed on 15 September 2025).

**Figure 4:**
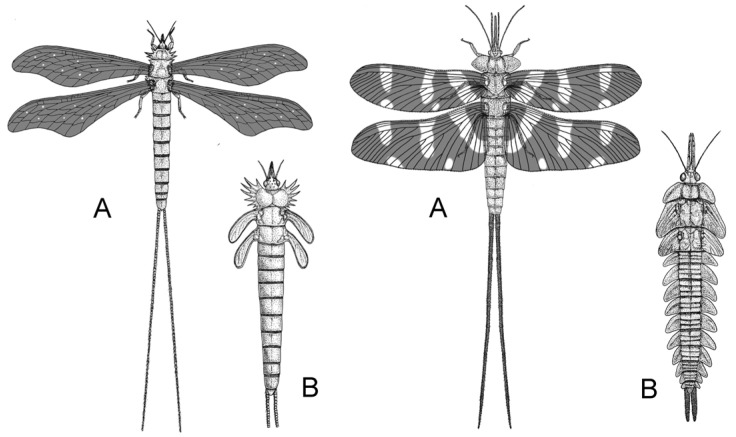
Palaeodictyopteroidea was an archaic superorder of palaeopterans, very diverse in Late Paleozoic, including some of the larger insects that flew on Earth. They represent the earlier radiation of herbivorous winged insects, already with specialized piercing-sucking mouthparts, which played a crucial role in plant reproduction during the Carboniferous and Early Permian. They declined in the Late Permian, likely with the new floral changes, which saw the rise of new feeders and a turnover of insect groups. (**A**) Winged adults and (**B**) nymphs with wing buds of the representatives of two different orders (modified from [49], CC BY 4.0; illustration by Martina Pecharová; via Wikimedia Commons: https://commons.wikimedia.org/wiki/File:Larvae_and_adults_of_Megasecoptera_and_Palaeodictyoptera.jpg (accessed on 15 September 2025)).

Critical innovations began in the Middle Devonian. Some Progymnospermopsida, already with gymnosperm-like anatomy but still reproducing with undifferentiated spores, evolved distinct male microspores and female megaspores, a crucial step toward the specialized heterospory of seed plants [11,38,39,40]. Early gymnosperms advanced further with the evolution of the ovule (Figure 3), in which a retained megaspore was enclosed by integuments, leaving a micropylar opening through which a nucellar exudate secreted a pollination drop that captured airborne pollen [8,9,41]. Once fertilization has occurred, this structure becomes a seed. Fossil evidence of such a mechanism is well documented in a Late Carboniferous Callistophytales, where a prepollen-filled exudate was preserved protruding from the micropyle, demonstrating a pollination-drop system likely ancestral in seed plants—first serving wind pollination and later co-opted for insect mediation [9,25,42]. Gymnosperms diversified during the Carboniferous, with early seed plants such as Medullosales and Callistophytales together with more advanced lineages like Cordaitales, radiating into trees, shrubs, and climbers [11,43,44]. While early spermatophytes were probably wind-pollinated, evidence from Upper Carboniferous medullosaceans suggests the earliest insect involvement: their large, heavy prepollen, enclosed in structures with glandular trichomes and fleshy tissues (Figure 3), likely offered nutritive rewards to large arthropod pollinators such as palaeodictyopterans [9] (Figure 4).

In the Permian, increasingly arid climates favored xeromorphic gymnosperms, and probable insect pollination is indicated in groups like Peltaspermales and Glossopteridales, the latter bearing flower-like female organs and producing abundant pollen and seeds suggestive of entomophilous adaptations [43,45,46].

These botanical shifts coincided with the first massive radiation of pterygote insects during the Carboniferous and Permian, likely driven by plant evolution [3,7,47,48].

Archaic palaeopterans were the first herbivorous insects that developed complex, modified mouthparts (Figure 4) and most likely played a crucial role in the reproduction of Carboniferous seed plants [7,9,14,31,47,49], together with early neopterans, represented by Polyneoptera and stem Paraneoptera.

**Figure 5:**
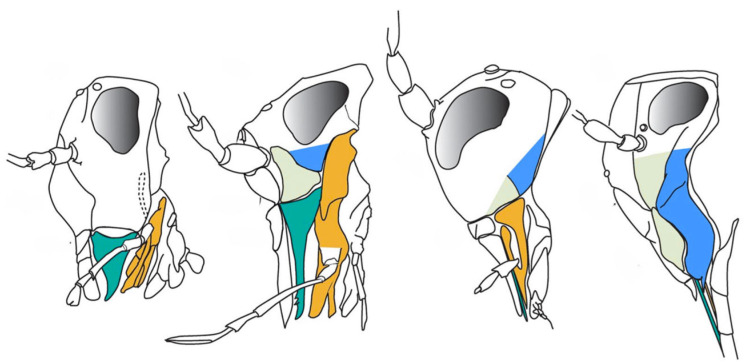
Schematic representation of head and mouthpart morphology in various Acercaria, illustrating the hypothesized evolutionary transition from basal chewing to advanced piercing–sucking mouthparts. From left to right: psocid with basal mandibulate mouthparts; permopsocid with elongate mandibles, intermediate between chewing and piercing–sucking; thripidan ground pattern with piercing–sucking mouthparts; and hemipteran with highly specialized piercing–sucking structures. Color code: mandible—green; maxilla—yellow; anterior part of gena (mandibular lobe)—grey; posterior part of gena (maxillary lobe)—blue (Modified from [52], CC BY 4.0; illustration by Thierry Bourgoin and Patricia Nel; via Wikimedia Commons: https://commons.wikimedia.org/w/index.php?curid=89689105 (accessed on 15 September 2025)).

The Permian saw the decline of palaeopterans [50,51] and the rise of neopterans such as early bugs, thrips, and early holometabolans (beetles, lacewings and stem mecopteroids), while Orthoptera and Grylloblattodea diversified among Polyneoptera [3,44,52]. These groups also evolved increasingly specialized mouthparts from basic chewing mouthparts (Figure 5): Acercaria developed piercing–sucking styles, and holometabolans produced the earliest siphonate, non-piercing proboscides, establishing the major mouthpart classes that persist today [3,52,53].

**Figure 6:**
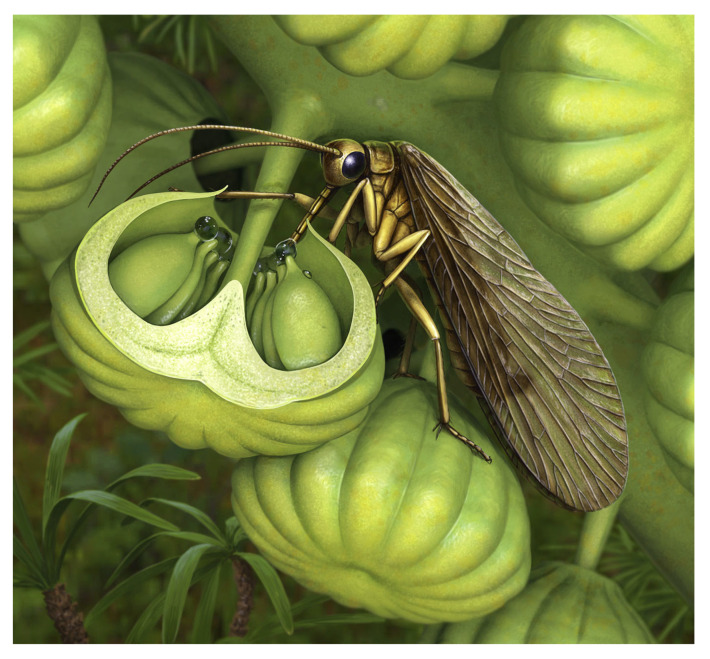
*Marimerobius* sp. (Protomeropidae) feeding through its proboscis on a *Permoxylocarpus* (Peltaspermales) cupula, figured dissected to reveal ovules with pollination drops. This species represents the earliest known example of non-piercing haustellate mouthparts in holometabolous insects, likely adapted for absorbing sugary fluids. Multiple fossils of *Marimerobius.* have been found in association with Peltaspermales pollen, suggesting the involvement of these insects in early gymnosperm pollination during the Lower Permian. Adapted with permission from [25]. Date: 2022; copyright owner: Aleksander V. Khramov; artist: Andrey Atuchin.

Exceptional insight comes from the Early Permian Chekarda Lagerstätte in Russia, which preserves over 290 insect species across 25 orders alongside diverse gymnosperms [7,54,55]. Fossilized gut contents show monospecific or mixed accumulations of pollen from Peltaspermales, Glossopteridales, and conifers, providing clear evidence of specialized palinophagy and intense insect–plant interactions [5,25]. Among the most remarkable insects is the genus *Marimerobius* (Fam. Protomeropidae), the earliest known holometabolan with a siphon-like proboscis adapted for fluid feeding, probably on pollination drops of Peltaspermales (Figure 6) [24,25].

Protomeropidae, early mecopteroids perhaps stem-Amphiesmenoptera (superorder which includes caddisflies, moths and butterflies), represent the first documented radiation of non-piercing long-proboscid nectar feeders, precursors to later specialised Mesozoic guilds, and likely went extinct with the climatic upheavals of Pangea [24,56].

By the Late Permian, insect faunas remained diverse, and analyses indicate no catastrophic family-level extinction at the Permian–Triassic boundary. Instead, the Early Triassic retained about two-thirds of Late Permian families, with diversity loss attributed more to slowed diversification than to wholesale extinction [51,57]. Even so, the transition marked a profound restructuring of insect assemblages, as dominant Paleozoic groups waned and a more modern, “post-Paleozoic” fauna began to take shape [50].

### 2.3. The Mesozoic Third Phase: Advanced Gymnosperms and Pollinator Guilds

The Mesozoic represents a fundamental restructuring of plant-insect relationships, driven by the Permian-Triassic extinction. It was characterized not by simple recovery, but by the rise of advanced gymnosperms and the evolution of sophisticated, often specialized, pollination mutualisms with insects—a complex ecological world of pollinators that flourished for over 100 million years before the dominance of angiosperms.

This evolutionary phase is defined by three major developments, described in the subsequent paragraphs: (1) the P–T extinction as a catalyst wiping out Paleozoic ecosystems and creating the ecological vacuum and evolutionary opportunity that allowed for a new phase to begin; (2) the rise of gymnosperms as advanced plants with complex reproductive structures and rewards (fleshy tissues, sugary pollination drops) to attract insects; and consequently (3) a pre-angiosperm pollination revolution, i.e., a spectacular radiation of pollinator guilds that engaged in specialized, often obligate, relationships with gymnosperms.

#### 2.3.1. The Permian-Triassic Crisis and Its Aftermath

The Permian–Triassic (P–T) crisis, the most severe extinction in Earth’s history, eliminated ~95% of species and about half of marine and terrestrial families [58,59]. On land, forests collapsed, producing a global “coal gap” lasting nearly 10 million years, with palynological assemblages dominated by pioneer pteridophytes [60,61,62,63,64]. Some authors, however, argue that floral losses may be overstated due to taphonomic biases and localized refugia [65,66,67].

Insects show a similar pattern: diversity dips in Early Triassic deposits may reflect faunal replacement or preservation bias rather than true extinction [12,51,68,69]. Nonetheless, the P–T transition marks a major evolutionary turnover, with the extinction of Paleozoic orders such as Palaeodictyopteroidea and the rise of holometabolous lineages, including the appearance of new orders, such as Diptera, Hymenoptera, and Lepidoptera [1,5,48,70]. The Triassic records a progressive recovery, from Early Triassic faunas dominated by survivors from the end of the Permian to Late Triassic assemblages comparable to those of the Early Jurassic [50,71]. Although direct evidence such as pollen-filled gut contents is absent, indirect data indicate the establishment of new insect–gymnosperm pollination mutualisms, which radiated in the Early Jurassic and underpin the diversification of pollination systems still evident today [5,6,69].

#### 2.3.2. Floristic Recovery and the Rise of Mesozoic Gymnosperms

Starting in the early Middle Triassic, global vegetation began to recover [64]. This recovery was not a simple return to the Paleozoic status quo but a fundamental restructuring of plant communities. Early gymnosperms, such as Lyginopteridales, Medullosales, and Callistophytales, were already extinct in the Early Permian. Others, like Cordaitales, Glossopteridales, Peltaspermales and Lebachiales, did not cross or only briefly survived the P-T boundary [11,72,73].

In their place, a new flora emerged. Late Paleozoic survivors like Cycadales, Ginkgoales, Pinales, and Gnetales became fundamental components of Mesozoic ecosystems, alongside groups that originated in the Late Permian, such as Bennettitales and Czekanowskiales [11,74]. They were joined by new groups that radiated during the Triassic and Early Jurassic, including Voltziales, cupressoid and Cheirolepidiaceae conifers, Pentoxylales, and Caytoniales [11,72].

This Mesozoic flora, in terms of classes, represented the peak historical diversity of insect-pollinated seed plant lineages. These included Cycadopsida, Pinopsida, Bennettitopsida, Gnetopsida, possibly Ginkgoopsida (if Czekanowskiales are included) and Angiospermopsida, compared to only three extant classes (Cycadopsida, Gnetopsida, and Angiospermopsida) with major insect-pollinated members (Figure 1). This vast diversity of gymnosperms began to decline in the mid-Cretaceous with the rise of angiosperms. Today, only Cycadales, Pinales (though excluding many Mesozoic families like Cheirolepidiaceae), Ginkgoales (a single species), and Gnetales remain as survivors [6,9,11,72].

Pollination systems of mid-Mesozoic gymnosperms were diverse but can be broadly grouped into two categories based on the reward offered and the corresponding insect pollinators: (a) the pollen and tissue reward systems; and (b) the pollination drop and nectar reward systems.

(a) The pollen and tissue reward systems primarily included plants with compact cones that produced abundant pollen and offered fleshy tissues as a reward. These structures were typically visited by small, mandibulate (e.g., beetles) or with piercing-sucking mouthparts (e.g., thrips), insects that lived in close association with the reproductive organs, often as larvae feeding within the cone tissues [6,9,75,76].

Evidence of this association, in the form of tunnelling damage and coprolites in strobili likely caused by beetle larvae, dates back to the Late Triassic and is known throughout the Mesozoic in plants like cycads, Bennettitales, and Pentoxylales [23,32,76,77]. Compelling evidence comes from a well-preserved, likely a cucujid beetle larva discovered inside the fleshy fructifications of a Cretaceous Pentoxylales, confirming beetle consumption of seed plant reproductive structures prior to angiosperm dominance [78].

Cycadales are the most ancient seed plants living today, although their modern diversity is the result of a recent radiation in the Miocene-Pliocene (12–5 Ma) or even later [79,80,81,82]. Actually, a mutualism involves beetles (mainly weevils and cucujids) that consume pollen and cone tissues while acting as pollen vectors, involving highly specific and complex interactions [32,79,80]. Coleoptera and Cycadales have both a very long evolutionary history [44], and their mutualist association is thought as an Early Mesozoic heritage [7,9,76,81,82]. However, although there is evidence of associations between cycads and beetles dating back to the Mesozoic [23,44,83], cycad-specific lineages of modern weevils—currently the most important and diverse host-specific pollinators of extant cycads among beetles—are relatively young, and likely derived from a shift from angiosperm hosts to these plants [33,84]. Another modern guild involves primitive thrips (Aeolothripidae) restricted to pollinating the Australian genus *Macrozamia* [85,86,87]. While basal Thysanoptera likely played a broader role in Mesozoic gymnosperm pollination [6,81,86,87,88], the specific *Macrozamia*–thrips mutualism is also relatively young, probably less than 10 million years old [33,89].

**Figure 7:**
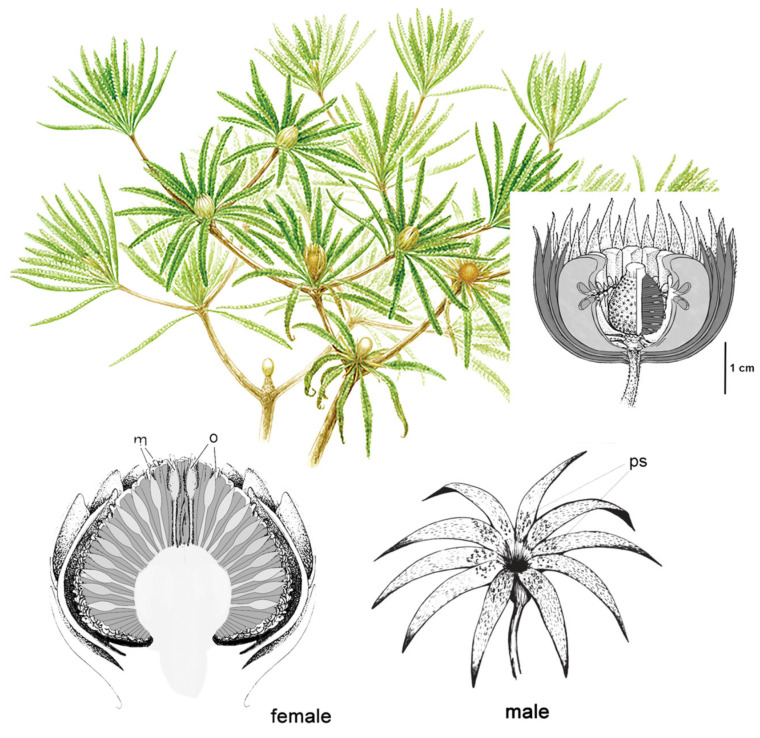
Bennettitales, Williamsoniaceae. (**Top**): restoration of the presumed monoecious shrub-sized *Wielandiella*, showing dichotomous branching and cones at various stages of maturation and senescence (Illustration by Pollyanna von Knorring, reproduced from [95], with permission; inset shows a reconstruction of the bisexual “flower” of *Williamsoniella*. (**Bottom**): schematic reconstruction of *Willamsonia* female cone ((**left**); cross section) and *Weltrichia* male “flower-like” strobilus (**right**) likely belonging to the same genus of dioecious plants ((**left**), original drawing; (**right**), illustration by Diana Silvia Guzmán-Madrid, CC BY 4.0, via Wikimedia Commons: https://commons.wikimedia.org/w/index.php?curid=11794915 (accessed on 15 September 2025)). Abbreviations: m, micropyles; mc, microsporangia; o, ovules; ps, pollination sacs.

Bennettitales (Figure 7) provide another quintessential example of this pollination mode. They produced highly complex cones, often with abundant bracts and, uniquely among gymnosperms, bisexual reproductive structures in several species [11,72]. Despite superficial similarity, they were not closely related to cycads [72,90]. Fossil evidence from both families (Cycadeoideaceae and Williamsoniaceae) shows tunnelling damage at the interface of ovulate and pollen organs, likely caused by beetle larvae [9,75,91]. Attractive structures like resin bodies together with robust woody tissues may have been adaptations to lure Coleoptera while limiting destructive feeding [92]. Cycadeoideaceae cones were hermaphroditic and remained closed at maturity, suggesting self-pollination, potentially aided by boring beetles [9,75,91,93]. Williamsonaceae bore open, flower-like structures that may have secreted sugary substances or volatiles to attract pollinators like beetles and true flies [94,95]. Features like long, arching bracts may have restricted access to ovules to insects with long proboscides [13,24]. Decoy mechanisms common in modern cycads (thermogenesis, volatiles) were likely present in Bennettitales and other Mesozoic gymnosperms [32,33,96] and were likely mimicked, in the Early Cretaceous, by certain basic angiosperms with large, showy flowers, similarly pollinated [6,93,97,98,99].

(b) The pollination drop and nectar reward systems involved a modification of the basal pollination drop, introducing higher levels of nutritional substances to produce an energy-rich reward for highly mobile, fluid-feeding insects [6,24,41].

**Figure 8:**
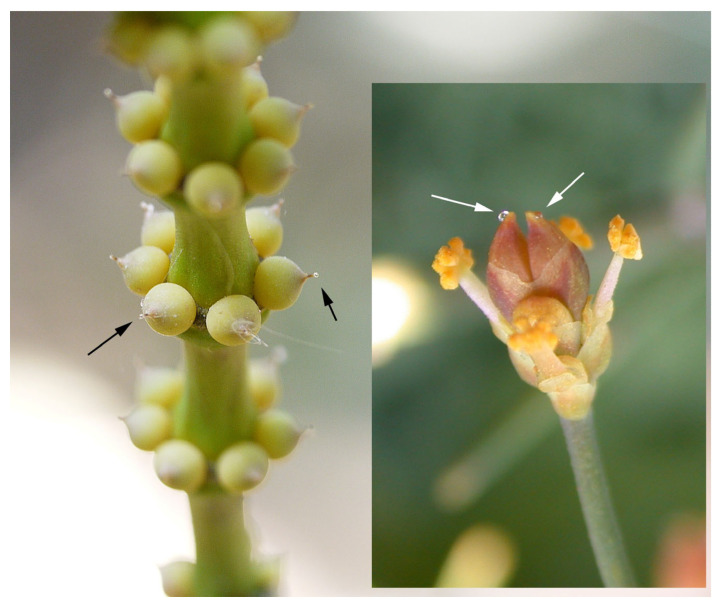
Gnetales. Female cones of *Gnetum scandens* (Gnetaceae) with pollination drops exposed at the micropylar openings (**left**), and male cone of *Ephedra foemina* (Ephedraceae) with pollination drops produced by sterile ovules at the distal centre of the cone (**right**). Arrows indicate the pollination drops. Left, image by Dinesh Valke, originally published on Flickr, licensed under CC BY-SA 2.0; license confirmed by FlickreviewR (24 September 2016) via Wikimedia Commons: https://commons.wikimedia.org/w/index.php?curid=51703376 (accessed on 26 December 2025); (**right**), image by Gideon Pisanty, licensed under CC BY 3.0, via Wikimedia Commons https://commons.wikimedia.org/w/index.php?curid=3933762 (accessed on 26 December 2025).

Extant Gnetales are the only group that has preserved this mechanism (Figure 8), secreting sugary drops (including on sterile ovules in male strobili) with nutritional levels comparable to angiosperm nectar, which lure visitors and ensure pollen collection; many Mesozoic lineages likely used similar tactics [9,24,41,100,101,102,103,104]. Unlike pollen/tissue-reward plants, this second category of gymnosperms often has loosely arranged unisexual organs, with notable exceptions such as Cheirolepidiaceae and other presumed insect-pollinated Mesozoic conifers bearing compact cones; funneled cone scales and abundant *Classopollis* pollen—frequently associated with insect fossils—strongly indicate entomophily within a largely wind-pollinated clade [6,9,72,105,106].

#### 2.3.3. Pre-Angiosperm Complex Mutualist Balances

This syndrome chiefly engaged pollinators with non-piercing haustellate mouthparts that evolved repeatedly in Holometabola [6,24,107,108,109,110,111]. Functionally, proboscides derive from maxillary/labial modifications and range from short, mobile sponges for superficial fluids (e.g., fly labellum) to long, tubular siphons for concealed rewards (e.g., butterflies, several bees, bombyliids flies), with associated flight and sensory adaptations [1,3,6,24,112,113,114,115,116,117,118]. A classic “Darwin’s race”—progressive concealment of rewards vs. proboscis elongation—promoted pollen contact [24,119,120].

Fossil records recognize two major waves of Mesozoic long-proboscis nectarivores: a mid-Mesozoic phase (Middle Triassic–Early Cretaceous) dominated by specialized Mecoptera, Neuroptera, and Diptera, and a mid-Early Cretaceous–Recent phase coincident with angiosperm radiation, which led to the pollinator guilds that we know today; Early Cretaceous overlap implies early interactions with flowering plants [6,22].

**Figure 9:**
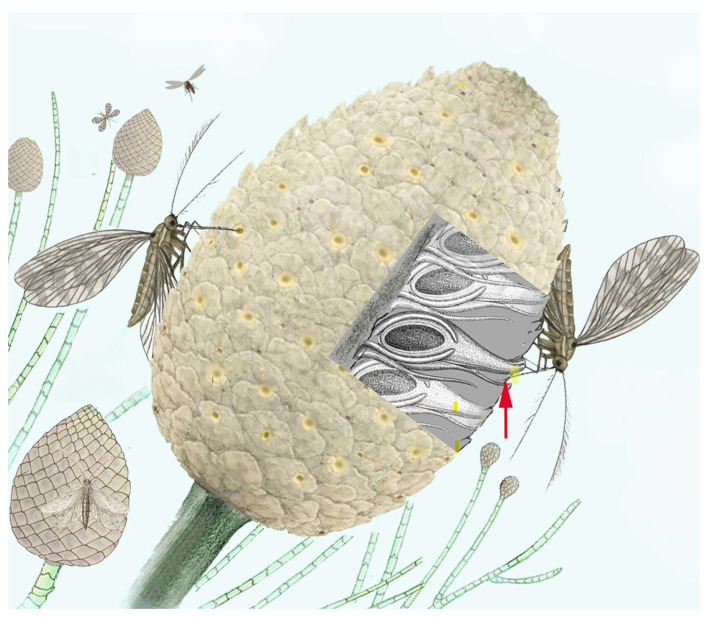
Reconstruction of a Lower Cretaceous plant-insect association between an entomophilous female cone of a cheirolepidiaceous conifer (*Alvinia*) and meropsychids, specialized anthophilous scorpionflies (*Vitimopsyche*), which acted as vectors of *Classopollis* pollen. The insects were likely attracted by the bright coloration of the funnel orifice and its sugary secretions. On the right, section of the cone showing ovule scales with the nectariferous funnel (indicated by the arrow) probed by the insect proboscis.

Before angiosperms, mid-Mesozoic fossils already show long-proboscid nectarivores matched to tubular gymnosperm organs and insect-type pollen, implying complex insect pollination predated flowering-plant dominance [6,7,9,20,22,23,24,30,88,106,121,122]. Cheirolepidiaceae exemplify this: funneled female scales guided small or long-proboscid insects to nectary-like secretions (Figure 9), and their *Classopollis* pollen is ubiquitous in Jurassic–Cretaceous deposits, often found in association with herbivorous insect fossils [6,9,72,105,106]. Extant Gnetales attract diverse short-proboscid or non-proboscid visitors (flies, midges, wasps, bees, beetles, thrips); Mesozoic gnetaleans likely ranged from extant-like forms to species with exceptionally long micropyles and bracteate/hairy tufts—potential adaptations to long, thin proboscides [6,10,101,102,104].

**Figure 10:**
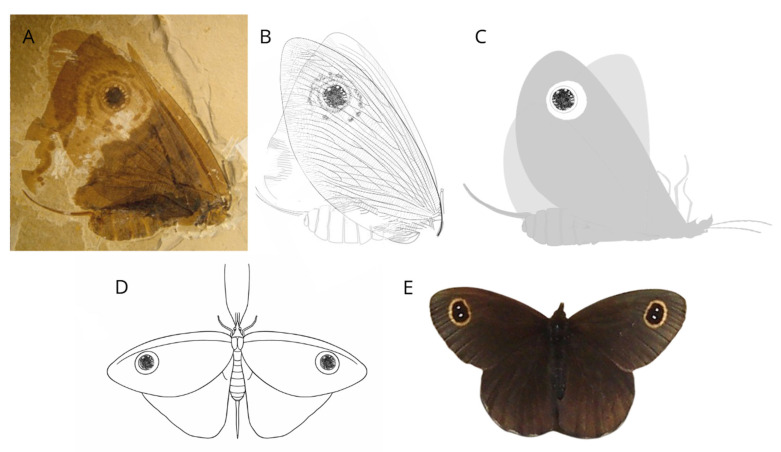
Kalligrammatidae (Neuroptera)—often referred to as the “Mesozoic butterflies”—represent a remarkable example of convergent evolution with modern Papilionoidea, which appeared more than 50 million years after their extinction. Their resemblance to modern butterflies was striking: large, colorful wings bearing eyespots and covered with scale-like structures, though anatomically distinct from those of Lepidoptera. Their elongated proboscides enabled them to feed on sugary secretions from the cones of entomophilous gymnosperms such as Bennettitales and Cheirolepidiaceae. The decline of these plant groups, coinciding with the expansion of angiosperms, likely contributed to the extinction of these extraordinary insects. (**A**) Fossil of the kalligrammatid *Oregramma illecebrosa*; (**B**) outline of fore- and hindwing with body; (**C**) reconstruction of the insect position during fossilization; (**D**) schematic restoration of the kalligrammatid species compared with (**E**) the modern butterfly *Erebia cyclopius* (Eversmann 1844). (**A**,**B**) adapted from [126] licensed under CC BY 2.0, originally published by BioMed Central Ltd. (London, UK); (**C**–**E**) original.

From the Upper Triassic to Upper Cretaceous, non-angiosperm mutualisms were in part already represented by groups that are now recognised as anthophilous (Thysanoptera, Coleoptera, Diptera). However, they were also dominated by groups that today have no or little importance as pollinators. Mecoptera (scorpionflies; Figure 9) and Neuroptera (lacewings) were much more diverse in the Mesozoic, developing very successful nectarivore lineages with long proboscis, some of which became popular for their incredible convergence with current butterflies (Figure 10) [6,9,20,24,30,123,124,125,126].

Large mandibulate Polyneoptera formed a now-vanished palynivory guild; Late Jurassic gut contents show Ensifera consuming Cheirolepidiaceae pollen, and gymnosperm pollen appears in some Phasmatodea and Embioptera, though regularity is uncertain [6,9,18,106,127,128,129]. Cretaceous ambers reveal anthophilous blattodeans with gymnosperm or early angiosperm pollen and floral parts associated [130]. Actually, Orthoptera and Blattodea are minor pollinators, whereas Phasmatodea and Embioptera are not known to consume pollen [6,131,132,133]. Within Acercaria, thrips and the archaic Permopsocida (with a mouth-cone intermediate between chewing and piercing–sucking; [134]) fed on pollen/nectar; Permopsocida vanished by mid-Cretaceous, likely displaced by angiosperm-associated pollinators [2,135,136]. An enigmatic order, Tarachoptera (Amphiesmenoptera), known only from Burmese amber, also failed to cross the Cretaceous [122,137,138].

Hymenoptera and Lepidoptera were less diversified and marginal until the mid-Cretaceous [6,9,22,24]. The oldest Hymenoptera are Middle-Triassic xyelid sawflies, and the earliest unambiguous Lepidoptera are Early-Jurassic micropterigid-like moths with mandibulate adults and primitive scales [1,68,109,139,140,141,142,143,144]. Through the Mesozoic, Hymenoptera diversified modestly until the mid-Cretaceous appearance of most modern groups, including the first bees [1,13,16]; apoditrysian Lepidoptera are absent before the Early Eocene, though basal Glossata occur earlier [24,145].

Amid these shifts, Diptera remained central from the Triassic onward and today are second only to bees in pollination importance; both Nematocera and Brachycera include long-proboscid anthophiles with flight/sensory traits suited to deep corollas [1,9,24,113,118,136,146,147,148,149,150,151,152,153,154]. The findings of Upper Jurassic flies with modern-like adaptations to anthophily were initially interpreted as evidence of the existence of angiosperms at that time [155]. However, since no remains of angiosperms in deposits older than the Early Cretaceous have ever been found, and *Classopollis*-type pollen were often found attached to the hairs of their bodies, it is more logical to deduce that nectarivore flies were already important pollinators in a world still devoid of flowering plants [6,7,9,118,156,157,158,159].

Finally, the Mesozoic sees the great radiation of beetles; larval feeding on gymnosperm sporophylls has been known since the Middle Triassic and persists in modern cycad mutualisms. Cretaceous ambers record a surge of plant-associated polyphagans, newly emerged groups related to the new flora, extinctions of gymnosperm-tied pollinators that failed to shift hosts, and at least one gymnosperm-angiosperm transition documented [22,23,26,44,70,76,98,121,160,161,162,163,164,165,166,167].

### 2.4. The Cretaceous Terrestrial Revolution: Angiosperm Radiation and the Evolution of Bees

Angiosperms (flowering plants) currently represent approximately 90% of all land plants in terms of species diversity and biomass [168]. They dominate nearly all terrestrial ecosystems and constitute a fundamental component of the modern food chain for numerous organisms, including humans. Their evolutionary success is often attributed to innovations in reproductive strategy compared to gymnosperms, most notably the enclosure of seeds within fruits that develop from the ovary after fertilization [15,90,169,170].

The fossil record provides robust evidence of an intense diversification and radiation of angiosperms during the late-Early to early-Late Cretaceous, beginning with the first unambiguous tricolpate pollens from the Barremian–Aptian (~121 ma) and floral assemblages from the Aptian (~115 ma), followed by an explosive increase in diversity during the mid- and late-Cretaceous (Aptian–Turonian, ~115–90 ma) [15,158,171,172]. Despite this evidence, the timing and mechanisms underlying the origin and early radiation of angiosperms remain a subject of vigorous debate—the Darwin’s “abominable mystery”—owing to conflicting interpretations of macrofossil versus microfossil data and discrepancies between paleontological and molecular datasets [169,173,174]. Molecular clock estimates often suggest pre-Cretaceous origins, ranging from the Late Carboniferous to the Middle–Late Jurassic, implying a long cryptic evolutionary history unrepresented in the fossil record [15,172,173,175]. However, such estimates may be inflated if they fail to account for dramatic accelerations in diversification rates during the angiosperm radiation [172].

What is clear is that angiosperms rose to ecological dominance during the Late Cretaceous, catalysing the Cretaceous Terrestrial Revolution (KTR), a profound restructuring of trophic networks that set the stage for modern terrestrial ecosystems [16,176,177]. A hallmark of this ecological shift was the co-diversification of flowering plants and their pollinators, most prominently bees. Although bees are today the most diverse lineage of palinivory insects and a vital component of terrestrial biomes worldwide [17,178,179,180,181], their evolutionary origins trace back to the mid-Cretaceous in close association with angiosperm expansion [1,5,13,15,16].

Bees are thought to have originated from wasp-like ancestors in western Gondwana (modern Africa and South America) in arid habitats similar to present-day biodiversity hotspots for these insects [17,178,181]. This evolutionary transition marked a profound ecological innovation, shifting from carnivorous brood provisioning with insect prey to a strictly herbivorous, anthophilous diet of pollen and nectar, a change tightly coupled to the concurrent rise and ecological expansion of angiosperms during the Cretaceous (~130–66 ma) [1,13,182,183]. A central morphological adaptation was the evolution of branched, plumose body hairs. Unlike the simple setae of their wasp ancestors, these hairs dramatically increased the surface area for pollen adherence and could attract airborne particles through electrostatic forces, making bees unparalleled pollen vectors [184,185,186]. Over time, these hairs were further specialized into compact pollen-transporting structures (scopae) on the hind legs or abdomen, culminating in the evolution of corbiculae (pollen baskets) in the corbiculate bee clade [187].

**Figure 11:**
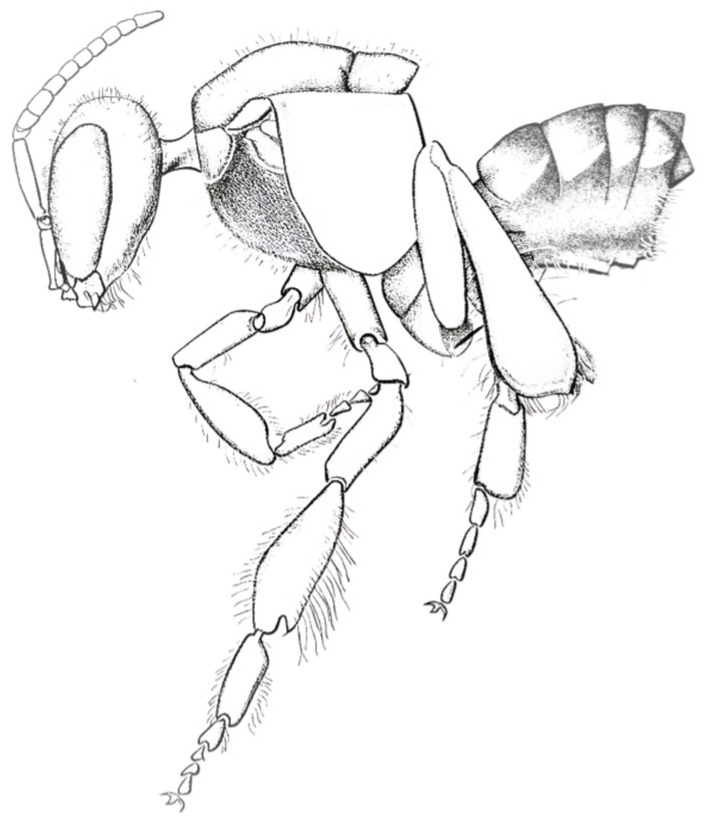
Original illustration based on descriptions and graphical reconstructions of *Cretotrigona prisca*, the earliest known fossil bee, discovered in Cretaceous (presumed Maastrichtian) amber from New Jersey, USA.

The fossil record, though sparse, corroborates this early diversification. The controversial *Melittosphex burmensis* from Burmese amber (~100 ma) exhibits a mosaic of bee-like traits (e.g., branched hairs) and wasp-like features, leaving its status as a stem bee or close apoid relative uncertain [188,189]. This, alongside other mid-Cretaceous transitional fossils and possible halictid nests, points to ongoing evolution. The oldest uncontested bee fossil, *Cretotrigona prisca* from New Jersey amber (~65 Ma; Figure 11), already belongs to the crown group Meliponini (Apidae), implying a much earlier, hidden diversification [190,191].

Phylogenetic and molecular data support an Early Cretaceous origin of Apoidea (~120 Ma), a process likely facilitated by the fragmentation of Gondwana beginning ~175 ma, which promoted geographic isolation and early lineage divergence [16,17]. By the end of the Cretaceous, all extant bee families were present.

This early diversification was further structured by major geological and climatic events. The continued separation of landmasses promoted endemism, while transient Tertiary land bridges facilitated dispersal into the northern hemisphere, where bees adapted to temperate floras [16,17]. Mass extinctions also played a formative role; the K–Pg event (~66 ma) and Eocene-Oligocene transition eliminated numerous taxa but created ecological opportunities for surviving clades to undergo adaptive radiations in tandem with their eudicot hosts [190,192]. Morphologically, early bees were likely short-tongued, a condition retained in basal families (Andrenidae, Colletidae, Halictidae). The increasing floral complexity of angiosperms later drove the evolution of long-tongued lineages (e.g., Apidae, Megachilidae), firmly anchoring bees in their mutualistic role and reshaping plant reproductive strategies [16]. Collectively, these geological, climatic, and biological factors forged bees into one of the most specialized and ecologically significant pollinator groups, whose coupled diversification with angiosperms during and after the Cretaceous Terrestrial Revolution fundamentally reshaped terrestrial ecosystems.

## 3. The Resilience of Plant–Pollinator Interactions: Lessons from Deep Time for the Anthropocene

Deep-time evidence suggests that pollination systems have demonstrated resilience through past environmental crises. Even profound environmental crises, such as the Cretaceous–Paleogene mass extinction, did not cause the collapse of entomophilous pollination but instead reshaped ecological networks. Generalist species often persisted and temporarily dominated, while specialists were disproportionately vulnerable [30,190,192,193,194]. This dynamic of disruption, survival, and reassembly illustrates the adaptive flexibility of plant–pollinator interactions—a theme that continues to resonate in the Anthropocene. Today, however, pollination systems face unprecedented pressures from climate change and human-driven environmental change. Urbanization, deforestation, agricultural intensification, and pollution are driving alarming declines in insect biodiversity and pollinator populations, with cascading consequences for ecosystem services and plant reproduction [195,196,197,198,199,200,201,202,203,204,205,206]. These stressors disrupt pollination dynamics and increase the risk of decline or extinction in plants dependent on specific pollinators. Yet, as in the deep past, such disruptions may also create opportunities for new associations. Empirical evidence demonstrates the capacity of networks to reorganize: in a century-scale study of a temperate forest understory community, 76% of original plant–pollinator interactions were lost and nearly half of historical bee species were extirpated, but extraordinary, novel interactions emerged, indicating a surprising degree of resilience and adaptability [207].

Adaptive responses are evident on both sides of the interaction. Among pollinators, foraging plasticity is a critical buffer against environmental stress. Generalist pollinators such as *Bombus terrestris* and syrphid flies expand their floral ranges when preferred resources decline, maintaining ecosystem function despite habitat fragmentation and phenological mismatches [195,196,197,208,209]. Urban studies similarly show bees incorporating non-native plants into their diets, allowing persistence in modified habitats [199,210]. However, specialists—such as oligolectic bees dependent on single plant taxa—remain highly vulnerable, heightening the risk of local extinctions and potential network collapse [196,211]. Plants, in turn, exhibit their own forms of plasticity, altering floral morphology, nectar production, scent, or phenology to attract alternative pollinators under stress [198,202,207,212,213].

Taken together, the evolutionary history of angiosperm–insect interactions illustrates both the creative power and the resilience of pollination systems. The Cretaceous angiosperm radiation was a critical turning point, driving the diversification of pollinators and promoting the coevolutionary traits that sustain modern ecosystems, with bees as their most prominent and emblematic representatives. At the same time, the persistence and reorganization of pollination through deep-time crises underscores the resilience and adaptive capacity of these systems. This long-term perspective offers crucial insights for the Anthropocene: while pollination networks are dynamic and capable of reassembly, their survival in the face of current unprecedented pressures will depend on conservation strategies that safeguard both pollinator diversity and the ecological conditions that enable such resilience.

## 4. Conclusions

The history of pollination in the Paleozoic and Mesozoic reveals a deep-in-time dynamic and evolving set of interactions between plants and insects. Since their earliest co-evolutionary stages, both groups developed adaptations that enabled intimate ecological relationships, laying the foundations for the complex pollination systems of today. These ancient syndromes were no less intricate or fundamental to the history of life on Earth.

Deep-time evidence shows that major ecological transitions—such as the rise of Mesophytic floras in the Permian and the Cretaceous Terrestrial Revolution with angiosperm expansion—drove more profound changes in insect diversity than the mass extinctions of the last 300 million years. These transitions opened new ecological opportunities, fueling adaptive radiations in both plants and pollinators. While this precedent suggests that current pollinator declines may also foster novel ecological arrangements, the unprecedented pace of human-driven environmental change may exceed the adaptive capacity of many species [195,196,211].

The Anthropocene thus presents both a challenge and an opportunity. Generalist pollinators and opportunistic plants may persist, but specialists risk functional extinction, creating gaps in pollination networks that may not be easily replaced [207,210]. Conservation strategies must therefore focus on safeguarding habitat diversity, genetic resources, and irreplaceable ecological roles, while research should track how networks reorganize in the face of loss.

Integrating deep-time insights with present-day challenges is essential for developing adaptive conservation strategies that foster both ecological stability and evolutionary resilience. Such an approach also offers a broader framework for understanding pollinator health and guiding sustainable management practices under accelerating environmental change.

## Data Availability

No new data were created or analyzed in this study.
